# Landscape Topography and Regional Drought Alters Dust Microbiomes in the Sierra Nevada of California

**DOI:** 10.3389/fmicb.2022.856454

**Published:** 2022-06-28

**Authors:** Mia R. Maltz, Chelsea J. Carey, Hannah L. Freund, Jon K. Botthoff, Stephen C. Hart, Jason E. Stajich, Sarah M. Aarons, Sarah M. Aciego, Molly Blakowski, Nicholas C. Dove, Morgan E. Barnes, Nuttapon Pombubpa, Emma L. Aronson

**Affiliations:** ^1^Division of Biomedical Sciences, University of California, Riverside, Riverside, CA, United States; ^2^Department of Microbiology and Plant Pathology, University of California, Riverside, Riverside, CA, United States; ^3^Center for Conservation Biology, University of California, Riverside, Riverside, CA, United States; ^4^BREATHE Center, University of California, Riverside, Riverside, CA, United States; ^5^Point Blue Conservation Sciences, Petaluma, CA, United States; ^6^Genetics, Genomics, and Bioinformatics Program, University of California, Riverside, Riverside, CA, United States; ^7^Sierra Nevada Research Institute, University of California, Merced, Merced, CA, United States; ^8^Department of Life and Environmental Sciences, University of California, Merced, Merced, CA, United States; ^9^Scripps Institution of Oceanography, University of California, San Diego, San Diego, CA, United States; ^10^Department of Geology and Geophysics, University of Wyoming, Laramie, WY, United States; ^11^Noctilucent Aviation, Bridgeport, TX, United States; ^12^Department of Watershed Science, Utah State University, Logan, UT, United States; ^13^Environmental Systems Graduate Group, University of California, Merced, Merced, CA, United States; ^14^Oak Ridge National Laboratory, Biosciences Division, Oak Ridge, TN, United States; ^15^Pacific Northwest National Laboratory, Biological Sciences, Richland, WA, United States; ^16^Department of Microbiology, Faculty of Science, Chulalongkorn University, Bangkok, Thailand

**Keywords:** aeolian processes, Asian desert, bacteria, biogeochemistry, dispersal, fungi, montane, provenance

## Abstract

Dust provides an ecologically significant input of nutrients, especially in slowly eroding ecosystems where chemical weathering intensity limits nutrient inputs from underlying bedrock. In addition to nutrient inputs, incoming dust is a vector for dispersing dust-associated microorganisms. While little is known about dust-microbial dispersal, dust deposits may have transformative effects on ecosystems far from where the dust was emitted. Using molecular analyses, we examined spatiotemporal variation in incoming dust microbiomes along an elevational gradient within the Sierra Nevada of California. We sampled throughout two dry seasons and found that dust microbiomes differed by elevation across two summer dry seasons (2014 and 2015), which corresponded to competing droughts in dust source areas. Dust microbial taxa richness decreased with elevation and was inversely proportional to dust heterogeneity. Likewise, dust phosphorus content increased with elevation. At lower elevations, early season dust microbiomes were more diverse than those found later in the year. The relative abundances of microbial groups shifted during the summer dry season. Furthermore, mutualistic fungal diversity increased with elevation, which may have corresponded with the biogeography of their plant hosts. Although dust fungal pathogen diversity was equivalent across elevations, elevation and sampling month interactions for the relative abundance, diversity, and richness of fungal pathogens suggest that these pathogens differed temporally across elevations, with potential implications for humans and wildlife. This study shows that landscape topography and droughts in source locations may alter the composition and diversity of ecologically relevant dust-associated microorganisms.

## Introduction

Each year, more than two billion metric tons of aerosolized soil particles (i.e., dust) are entrained into air currents and uplifted into the atmosphere of our Earth (Moulin et al., 1997; Perkins, 2001; Kellogg et al., 2004). Fine dust generation is expected to increase with droughts in source locations, leading to greater transport of material globally (Pu and Ginoux, 2017). One important—yet often overlooked—aspect of dust deposition is the functional role of far-traveled dust as a vector for microbial dispersal. Because microbial processes are important for regulating ecosystem functions (Bardgett et al., 2008; Mendes et al., 2015), in resource-limited montane systems, microorganisms and dust-associated nutrients have major effects on ecosystem functioning (Arvin et al., 2017). Therefore, it is essential to understand how abiotic factors like elevation or source-region drought affect dust-driven microbial dispersal and biogeography in montane systems.

Although air is often a vector of microbial dispersal (Bowers et al., 2009, 2011, 2012, 2013; Smith et al., 2013; Yooseph et al., 2013), the airborne environment can also be a true habitat for microorganisms capable of tolerating atmospheric conditions (Womack et al., 2010). Soil or root-associated fungal morphologies or life history traits, such as belowground mycorrhizal spore production, may determine the airborne dispersal ability and range of fungal taxa (Kivlin et al., 2014). For bacteria and archaea, motility and chemotaxis accelerate bacterial dispersal in porous environments, yet little is known about whether active cell dispersal confers similar advantages in airborne environments (Scheidweiler et al., 2020). Dispersal limitations or motility may ultimately dictate the functional capacity of resultant microbial communities in deposition zones.

Upon deposition, dust may reduce or eliminate nutrient limitations in aquatic ecosystems. For instance, dust promotes oceanic biological productivity by alleviating micronutrient deficiencies, such as iron (Fe), in the eastern equatorial Pacific (Winckler et al., 2016). Similarly, dust can serve as an exogenous subsidy to nutrient-poor terrestrial ecosystems such as slowly eroding ecosystems where slow rates of weathering limit nutrient supply from underlying bedrock. In a resource-limited montane ecosystem, Arvin et al. (2017) suggested that most of the growth requirement of native *Pinus jeffreyi* trees for the essential nutrient phosphorus (P) is dust derived. Additionally, microorganisms deposited with dust may support macronutrient bioavailability and plant uptake, and thus maintain vegetation community structure (Allen et al., 2003; Bever et al., 2010; Alguacil et al., 2011).

While exogenous inputs from dust can support biological productivity, they may also harm resident terrestrial biota. Fungal plant pathogens, such as those causing powdery mildew or sooty molds, infect a wide variety of plants, leading to both crop losses and forest blights (Peetz et al., 2007; Carisse et al., 2008). Dust storms and disturbance are linked to localized outbreaks of diseases caused by inhaling soil-dwelling fungal pathogens, such as *Coccidiodes* species, which causes valley fever (Gorris et al., 2018). Airborne chemicals, bacterial cells, and fungal spores within human lungs may produce damaging oxidative reactions (Samake et al., 2017). Metabolic and ecophysiological differences among microbial groups, including pathogenicity, underscore the importance of examining microbial diversity within airborne environments.

Environmental and temporal factors may differentially affect the composition and activity of airborne microbial communities (Griffin et al., 2002; Brodie et al., 2007). Airborne microbial interactions may expedite wet deposition and reduce particulate transit time. Shorter transit time may promote viability upon deposition, as it also limits airborne microorganisms’ exposure to environmental stress (Schlichting, 1964; Tesson and Santl-Temkiv, 2018). Yet, airborne microbes vary in their tolerance of stressful environmental conditions (Ehresmann and Hatch, 1975). For instance, cyanobacteria can withstand a wide range of atmospheric humidity, while microalgae prefer high humidity. Because cyanobacteria are highly resilient to airborne stress and produce compounds toxic to humans or animals (Carmichael and Li, 2006; Nielsen and Jiang, 2020; Wu et al., 2021) recent studies have concentrated on dispersal trajectories of this microbial group.

Although the consequences of microbial dispersal are vast, we know little about global vectors of microbial dispersal. Temperature gradients may stratify the airborne environment into distinct biogeographic zones and influence dispersal trajectories (Womack et al., 2010). Discrete events, like dust storms and wildfires, entrain particulate-transported microorganisms and biological ice-nucleating particles into air currents through convective columns (Kobziar et al., 2018; Kobziar and Thompson, 2020; Moore et al., 2021). Additionally, climatic and topographic features of depositional areas may filter dust-driven microbial transport into distinct terrestrial biogeographic zones.

Considering the potential implications of dust inputs for ecosystem structure and function, we evaluated the microbial dynamics and biophysical composition of dust along an elevation gradient on the western slope of the Sierra Nevada of California. To examine the effects of clinal variation on dust-associated microorganisms, we analyze incoming dust deposited at four study sites along an elevation gradient. This allows us to evaluate the relative contributions of drought, distance, and spatial features on biophysical properties of dust. We characterized dust microbiomes across two unusually dry years (2014 and 2015) to examine interactions among dust provenance and nutrient balance on microbial communities associated with incoming dust to the Sierra Nevada of California.

Our previous research found shifts in dust-driven nutrient inputs to this montane ecosystem. We demonstrated that the relative abundance of global (Asian) dust vs. regional (California’s San Joaquin Valley) sources of dust delivered to these study sites differed across elevations and varied between study years (Aarons et al., 2019). We also showed that sources of dust change throughout the dry season (Aciego et al., 2017), which is characterized by warm, dry summers with little to no precipitation from mid-May to October. Using isotope mixing models, we demonstrated that Asian dust inputs, combined with regional sources of dust, resulted in more heterogeneous dust materials.

**FIGURE 1:**
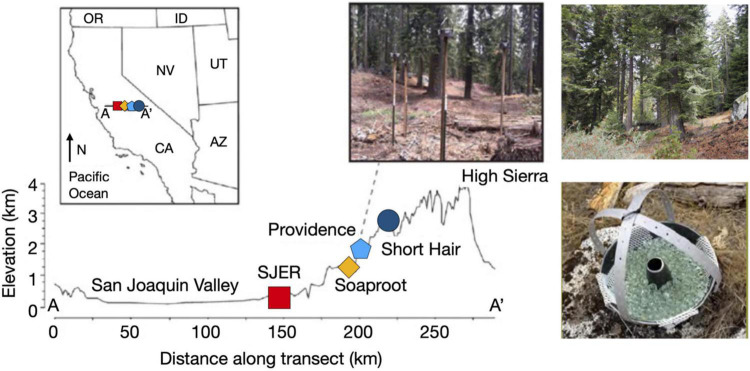
Map of sampling transect, dust collectors, and sites along the elevation gradient. Map illustrating sites from the San Joaquin Valley (low elevation; red square indicating SJER, 400 m elevation) to the High Sierra (high elevation; dark blue circle symbolizing Short Hair, 2,700 m). Inset image on the map of the state of California, United States illustrates sampling transect from A-A’ with sites (left to right, SJER, Soaproot 1,100 m, Providence 2,000 m, Short Hair) increasing with distance from the Pacific Ocean and rising in elevation. Dust collectors deployed at Providence, to the right of the inset map. Photograph of the highest elevation site at Short Hair is shown on the top right. Photograph of the dust collector, composed of a Teflon-lined Bundt pan filled with glass marbles and lined with Kevlar mesh shown at the bottom right (adapted from Aciego et al., 2017 and Aarons et al., 2019).

In this complementary study, we aimed to determine whether the composition of dust-associated bacterial and fungal community structure and function also differ across elevations and in response to droughts in global and regional dust source areas. The observed patterns in dust heterogeneity may correspond with increasingly intricate dust microbiomes, such as more phylogenetically or functionally diverse microbial communities. Deposited dust mixtures containing substantial contributions from both Asian and Californian dust sources may not only be more chemically complex than dust originating from one source region, but they may also be more complex microbially. Our study coincided with droughts in both California (Asner et al., 2016) and Asia (Zhang and Zhou, 2015), which increase dust emissions (Pu and Ginoux, 2017) and microbial entrainment in emitted dust (Kellogg et al., 2004), thus contributing more fine particulates and biological materials to these complex dust mixtures. Interannual variation in the location and severity of drought events may drive compositional differences in microbial community structure and the distribution of bacterial and fungal functional groups within dust deposited along this elevation gradient in the Sierra Nevada of California (Figure 1, modified from Aciego et al. (2017), Aarons et al. (2019) and Supplementary Table 1). We hypothesized that microbial diversity will be greater at higher elevations rather than at lower elevations, owing to more complex dust from multiple sources being deposited at higher elevations. We further hypothesize that these diversity trends will be more pronounced early in the dry season, and attenuate over time because regional soils from California’s San Joaquin Valley may be drier later in the dry season, and therefore aerosolize more fine dust from regional sources which will deposit across all elevations. Furthermore, we hypothesize that more dust-associated pathogens will be found at lower elevations, due to greater human-induced disturbance.

## Results

### Influence of Dust Heterogeneity and Elevation on Microbial Communities

We found that dust deposition to the Sierra Nevada study sites varied in proportions of dust originating from Asian and San Joaquin Valley sources (Aciego et al., 2017; Aarons et al., 2019). Our metric of dust heterogeneity (related to the proportion of Asian dust), calculated from concentrations of strontium (Sr) and Sr isotopic compositions (^87^Sr/^86^Sr) in San JoaquinValley and Asian Desert dust endmembers (as per Ingram and Lin, 2002; Yokoo et al., 2004) peaked when contributions of Asian dust approached those of San Joaquin Valley dust. While we used Sr concentrations and Sr isotopic compositions to calculate the metric of ranked dust heterogeneity, we also calculated neodymium (Nd) isotopic concentrations for use in determining dust provenance. Comparing elemental concentrations and radiogenic Sr and Nd isotope compositions, we quantified the role of regionally vs. globally supplied dust on microbial community composition across the elevation gradient. Dust heterogeneity, as reported from 2015, includes isotopic data to pair with all dust samples collected that year. When we reported dust heterogeneity from both years, we used paired isotopic analyses for samples in both July and August 2014; however, we used September 2014 dust samples as a proxy for October 2014 samples to coincide with the subsequent molecular analyses of dust collected during that sampling month.

**FIGURE 2:**
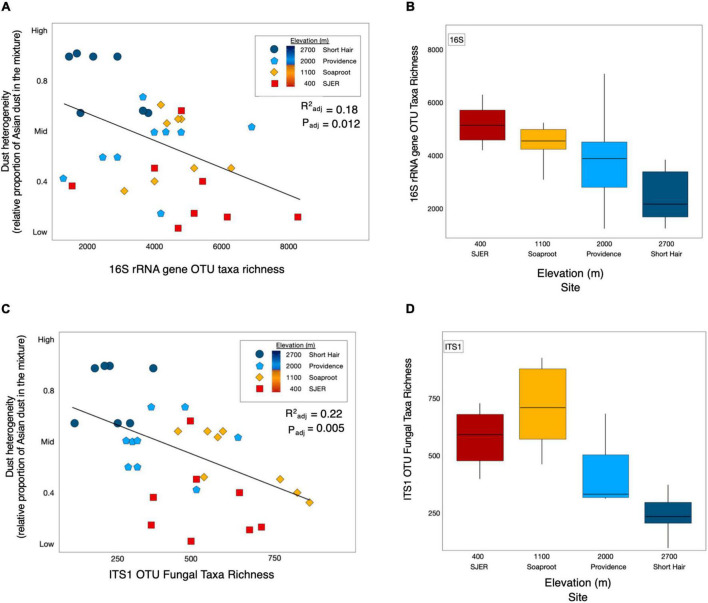
Dust heterogeneity varies by elevation and 16S rRNA gene OTU bacterial and fungal ITS1 microbial taxa richness. Dust complexity and 16S rRNA gene OTU microbial taxa richness varied by site along the elevation gradient. Linear regression of bacterial and archaeal taxa richness from 16S rRNA marker gene targeted amplicon sequencing shows that microbial taxa richness corresponded with dust heterogeneity and elevation **(A)**. Microbial taxa richness varies by site along the elevation gradient **(B)**. ITS1 fungal OTU taxa richness **(C)** was correlated with dust heterogeneity **(C)** and elevation **(D)** across sites in the study.

Using this metric, we found that dust heterogeneity positively correlated with elevation (p_*adj*_ < 0.001, *R*^2^_*adj*_ = 0.542). The dust at the lowest elevation site, San Joaquin Experimental Range (SJER; 400 m), was significantly less heterogeneous than dust deposited to the other sites. In contrast, dust detected at the highest elevation site, Short Hair (2,700 m), was more heterogeneous than at any other site. As dust heterogeneity increased with elevation, both bacterial and archaeal (hereafter, bacterial; p_*adj*_ = 0.010, *R*^2^_*adj*_ = 0.178; Figure 2) and fungal (*p*_*adj*_ = 0.005, *R*^2^_*adj*_ = 0.221; Figure 2) taxa richness declined. More heterogeneous dust at higher elevations corresponded to lower 16S rRNA gene microbial and ITS1 fungal taxa richness; likewise, less heterogeneous dust at lower elevations harbored greater 16S rRNA gene microbial and ITS1 fungal taxa richness. No effects of dust weights (m * g) were found for microbial richness or diversity (fungal taxa richness: *p* = 0.559; fungal alpha diversity: *p* = 0.650; bacterial taxa richness: *p* = 0.193; bacterial alpha diversity: *p* = 0.410); likewise, dust weights were not correlated with sampling duration.

We calculated alpha diversity with the Shannon-Wiener index, using rarefied OTU counts and detected no interannual differences (Supplementary Figure 1); bacterial counts were rarefied to 34,559 counts and fungi were rarefied to 7,302 counts using the “rrarefy” function from the “vegan” package (Oksanen et al., 2012). Although fungal alpha diversity differed by elevation (*p* < 0.001), elevation had no effect on bacterial alpha diversity (*p* = 0.412; Supplementary Figure 2).

No differences in bacterial alpha diversity were found by either month (*p* = 0.119) or year (*p* = 0.634; Supplementary Figure 1). Moreover, we found no overall temporal effects of sampling year (*p* = 0.413) or sampling month (*p* = 0.159) on fungal alpha diversity (Supplementary Figure 1). Apart from Short Hair, bacterial diversity was equivalent between years within each site (SJER *p* = 0.694, Soaproot *p* = 0.627, Providence *p* = 0.737); bacterial diversity at Short Hair was higher in 2015 than in 2014 (Supplementary Figure 2; *p* = 0.020). For fungal diversity, no interannual variation was detected at SJER (*p* = 0.732), Soaproot (*p* = 0.545), or Short Hair (*p* = 0.147), but we found higher fungal diversity in 2014 than in 2015 at Providence (*p* = 0.009).

**FIGURE 3:**
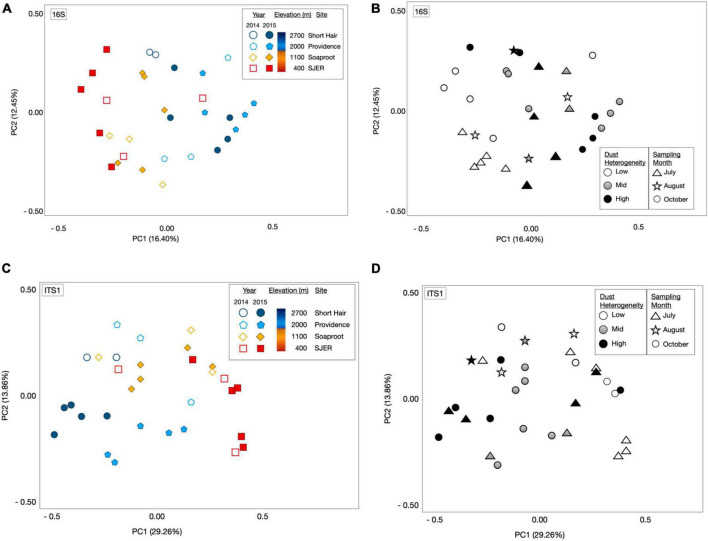
Microbial community composition shifts by elevation and sampling date. Principal coordinate analyses (PCoA) of 16S rRNA gene bacterial OTU microbial community composition revealed from targeted amplicon sequencing of marker genes **(A)** and the amplicons of the fungal ITS1 **(C)** region illustrated structural shifts by elevation and sampling year, as well as dust heterogeneity and sampling month **(B,D)**. Microbial communities represented by either open or filled shapes, corresponding to dust sampling year; two California drought years (2014 and 2015) and one Asian drought year (2015). 2014 dust microbiomes **(A)**, shown as open shapes with outlines colored by site); 2015 color filled shapes **(A)** with black outlines; colors corresponded to sites (as per legend, and on Figure 1: Map). Bacterial [16S rRNA marker genes; **(A)**] and fungal communities [ITS1 region; **(C)**] varied across elevation and sampling year. Dust bacterial **(B)** and fungal **(D)** communities in greyscale, ranked by dust heterogeneity (Low = white; Mid = gray; High = black), which was related to the proportion of Asian dust in samples deposited in California’s Sierra Nevada. Symbols in **(B)** (bacterial communities) and **(D)** (fungal communities) represented sampling months, with triangles for July, stars for August, and circles for dust samples collected in October.

**FIGURE 4:**
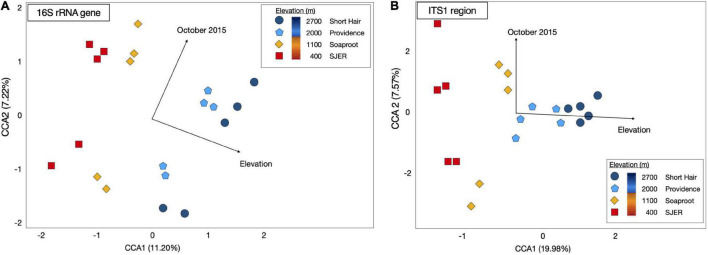
Canonical Correspondence Analysis (CCA) for dust-bacterial 16S rRNA targeted amplicons from marker genes **(A)** and ITS1 region amplicons for dust-fungal **(B)** microbiomes. Elevation and October sampling date contributed to structuring microbial communities. Dust-associated bacterial (16S rRNA marker gene amplicons) communities from 2015 were colored by site [colored symbols, **(A)**]. Dust fungal communities (ITS1) from 2015 were shown in **(B)**. Both fungal and bacterial communities were structured by elevation and the October 2015 sampling date, as evidenced by arrows representing the direction of the effect; arrow length corresponded to the magnitude of the effect of these environmental or temporal variables. CCA1 for dust-associated bacteria represented 11.20% of the variation; CCA2 for these bacterial 16S rRNA OTUs represented 7.22% of the variation; CCA1 for dust-associated fungal ITS communities represented 19.98% of the variation; CCA2 for dust fungal amplicons represented 7.57% of the variation in the composition of these communities.

Using PERMANOVA, we characterized compositional shifts and found that both fungal (p_*adj*_ ≤ 0.001) and bacterial (p_*adj*_ = 0.005) community composition varied along with the heterogeneity of deposited dust (Figure 3). Both bacterial community composition (p_*adj*_ = 0.001) and fungal composition shifted significantly by elevation (p_*adj*_ < 0.001). Elevation also significantly affected microbial communities at phylum and class levels, with significant effects on the composition of bacterial phyla (p_*adj*_ = 0.003) and fungal phyla (p_*adj*_ = 0.002), as well as both bacterial classes (p_*adj*_ = 0.001) and fungal classes (p_*adj*_ = 0.001). Additionally, when we restricted our analyses within specific sites, both fungal and bacterial community composition shifted annually (Bacteria *p* = 0.004; Fungi *p* < 0.001) and monthly (Bacteria *p* = 0.017; Fungi *p* = 0.001). Using Canonical correspondence analyses (CCA), we also found that elevation was a significant driver of fungal community composition during both years, and of bacterial community composition during 2015 (Figure 4). The relative abundance of fungal and bacterial classes varied over the course of the study (Supplementary Figure 3).

### Influence of Abiotic Factors and Dust Chemistry on Microbial Communities

Dust P concentrations were positively correlated with elevation during 2015 (*p* < 0.001, *R*^2^ = 0.61), but not in 2014 (*p* = 0.880, *R*^2^ = 0.003). In 2015, PERMANOVA analyses revealed that both fungal and bacterial community compositions were correlated with dust P (Bacteria *p* = 0.004; Fungi *p* = 0.001). Additionally, we found significant effects of dust sulfur (S) concentrations on fungal community composition (*p* = 0.005).

Fungal (*p* ≤ 0.001) and bacterial (*p* ≤ 0.001) community compositions were significantly related to dust chemistry (concentrations of elements or radiogenic isotopic compositions). Secondarily, of all the sampling months, the latest sampling date (October) in 2015 had a strong effect on structuring both fungal and bacterial communities (Figure 4).

PERMANOVA analyses revealed that isotope ratios used in determining dust provenance—Sr and Nd—were also correlated with both fungal (^143^Nd/^144^Nd, *p* < 0.001; *^87^Sr/^86^Sr, p* = 0.001) and bacterial (^143^Nd/^144^Nd, *p* < 0.001; *^87^Sr/^86^Sr, p* = 0.002) community composition. All Nd isotope compositions are reported hereafter as ε_Nd_, which is defined as ε_Nd_ = ([(^143^Nd/^144^Nd)_sample_/(^143^Nd/^144^Nd)_CHUR_]) −1 × 10^4^, where ^143^Nd/^144^Nd)_CHUR_ is the Nd isotopic composition of the Chondritic Uniform Reservoir (CHUR; Jacobsen and Wasserberg, 1980).

### Early vs. Late Dry Season Dust

**FIGURE 5:**
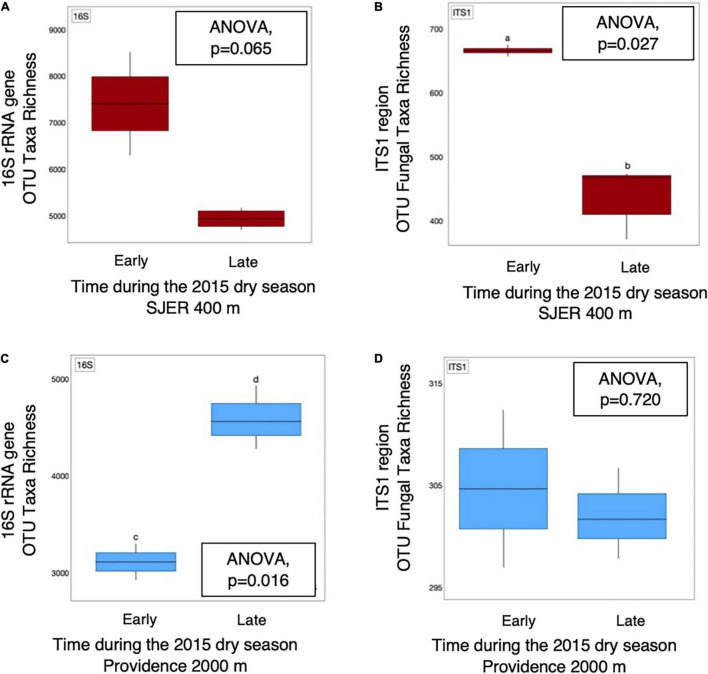
OTU taxa richness of dust microbial communities detected from 16S rRNA marker genes and amplicons from the fungal ITS1 region varied throughout the 2015 dry season. Bacterial **(A)** taxa richness was equivalent, with marginal differences detected, at SJER (400 m), the lowest elevation site throughout the 2015 dry season. At the same site, fungal **(B)** taxa richness was significantly higher in July (early in the dry season), than late in the dry season (in October). In contrast, at Providence (2,000 m), the second highest site, dust bacterial **(C)** taxa richness was significantly lower early in the dry season, than later during that same dry season. Dust fungal richness **(D)** was equivalent at Providence throughout that same time period.

We examined presence-absence data to calculate bacterial and fungal taxa richness and found that at the lowest elevation site (SJER; 400 m), fungal taxa richness was higher earlier (July) than later (August and October) in the 2015 dry season (*p* = 0.027; Figure 5). At SJER, bacterial richness exhibited a similar, marginally significant, trend (*p* = 0.065). In contrast, at Providence (2,000 m)—the second-highest elevation site—bacterial taxa richness was lower earlier in the 2015 dry season and increased later (*p* = 0.017); fungal taxa richness was statistically equivalent during the same time period (*p* = 0.720; Figure 5). We compared dust microbial diversity at each site between early and late dry season sampling periods and found that bacterial diversity was higher earlier in the dry season at the two lowest elevation sites (SJER, *p* = 0.03; Soaproot, 1,100 m, *p* = 0.03). Although dust from Short Hair (2,700 m) was the most heterogeneous, dust microbial richness and diversity did not vary across the dry season at this site for either fungi or bacteria.

We evaluated microbial community composition in dust collected in the early and late dry season via PERMANOVA analyses and detected significant compositional shifts in fungal community structure later in the dry season (*p* = 0.017; Supplementary Figure 3). Additionally, we detected a marginally significant interaction among elevation and fungal classes (p_*adj*_ = 0.008). In 2015, we found that the relative abundance of Arthoniomycetes (composed of ascomycete cup fungi with bitunicate apothecia) was higher in early dry season dust than in later-season dust (Supplementary Figure 4). In contrast, the relative abundance of fungi from Cystobasidiomycetes (a group of simple-septate basidiomycete fungi from the Pucciniomycotina) was lower earlier in the 2015 dry season than later (Supplementary Figure 4). Although there was a significant interaction between sampling year and early or late dry season dust for the relative abundance of bacterial classes (p_*adj*_ = 0.022), no differences were detected for bacterial phyla (p_*adj*_ = 0.353). However, the relative abundance of Acidobacteria was higher earlier than later in the 2015 dry season (*p* = 0.031; Supplementary Figure 4). Likewise, the relative abundance of Cyanobacteria (*p* = 0.001) was also higher earlier than later in the dry season (*p* = 0.001; Supplementary Figure 4).

### Variation in Fungal Functional Groups

**FIGURE 6:**
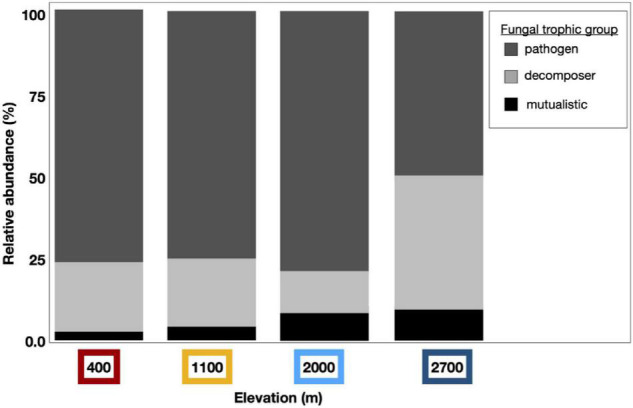
Dust fungal ITS1 trophic groups vary by elevation. Dust-associated fungal OTUs were assigned to functional groups using the online application FUNGuild; Those fungal taxa with high confidence levels were curated and binned by trophic group to pathogens (dark gray), decomposers (light gray), or mutualistic fungi (black). Bar graphs represent the relative abundance of these trophic groups within assigned fungal taxa from each elevation. The relative abundance of mutualistic fungal taxa increased clinally by elevation and more fungal pathogens were found at lower elevations than at the highest elevation site.

We detected compositional shifts across elevation in fungal mutualists (*p* < 0.001, as in lichen-forming or mycorrhizal fungal mutualists), fungal pathogens (*p* < 0.001), and saprotrophic fungi (i.e., decomposers, *p* < 0.001). Across sampling years, we detected compositional shifts in fungal mutualists (*p* < 0.001) and decomposers (*p* < 0.001). Sampling month (*p* = 0.029) significantly shifted the community structure of fungal mutualists. Along with these observed compositional shifts, the diversity of fungal mutualists significantly increased incrementally with increasing elevation (i.e., clinally; Figure 6 and Supplementary Table 2). The relative abundance of fungal mutualists increased with elevation (*p* = 0.002), with a greater abundance of mutualistic fungi at higher elevation than at lower elevation sites.

Interactions between elevation and sampling month were correlated with richness (*p* = 0.040), diversity (*p* = 0.050), and relative abundance of fungal pathogens (*p* = 0.042). The relative abundance of fungal pathogenic and decomposer taxa varied temporally, with interactions by sampling year and month (pathogens, *p* = 0.039; decomposer, *p* = 0.026). Indicator species analysis yielded a total of 22 significant taxa across all elevations, with a majority (15) of these found at Short Hair, the highest elevation site (Supplementary Table 3).

## Discussion

Our results suggest that dust-associated microbial communities in the Sierra Nevada of California are correlated with the chemical composition of dust and the environmental conditions in dust-source locations or recipient ecosystems. Overall, the differences in these dust-associated microbial communities appear to have corresponded to persistent drought conditions in California during our 2-year study, as well as the emergent drought in Asia in year two only, affecting the heterogeneity of dust deposited in both years. For the Asian drought year, dust heterogeneity (as related to the proportion of Asian dust in the mixtures) increased throughout the dry season. The competing droughts in Asia and California may have enhanced microbial diversity at lower elevations, while hindering dust-driven microbial inputs to highest elevations if dust-associated microorganisms were released during long-distance transport. These findings suggest that droughts in source locations may influence long-distance dispersal trajectories and the composition of both dust-bacterial and fungal communities in the Sierra Nevada.

### Dust Heterogeneity and Microbial Structure

Despite the expected finding of more heterogeneous dust at higher elevations, surprisingly, these dust mixtures harbored fewer microbial taxa than less-heterogeneous dust. Based on this finding, we rejected our hypothesis that more heterogeneous dust would harbor greater microbial taxa. Fewer detectable taxa in more heterogeneous dust suggests that microorganisms sorbed to dust particles traveling from Asia may have been jettisoned during the long-distance journey (Tesson and Santl-Temkiv, 2018). Although DNA from inviable organisms may still be detected by our molecular analyses, microbial viability may have been hindered in transit or prior to deposition, impacting the long-distance dispersal ability of a subset of viable dust microorganisms (Kellogg et al., 2004). Along with a reduction in San Joaquin Valley dust inputs, we found a corresponding decrease in the microbial richness within more heterogeneous dust mixtures.

Although we predicted that these diversity trends would attenuate over the dry season as higher elevation sites became progressively hotter and drier, we found varying richness and diversity patterns across elevations, years, and microbial groups. For instance, at the second highest elevation site, bacterial richness peaked late in the 2015 dry season, which may have resulted from more favorable site conditions. In contrast, fungal diversity peaked early in the dry season at the two lower elevation sites (SJER and Soaproot), with greater fungal taxa richness early in the 2015 dry season at SJER. Further, we found that dust microbial diversity and richness did not vary throughout the dry season at the highest elevation site (Short Hair). During this study, dust from Short Hair was characterized by low fungal and microbial diversity, which may have been related to cold temperatures or inhospitable conditions in this high elevation site. Thus, our hypothesis that dust microbial diversity trends would be more pronounced early in the dry season was partially supported, but only at our two lowest elevation sites that harbored the least heterogeneous dust.

### Drought and Dry Season Impacts

Human activities, such as irrigation and farming, may alter seasonal edaphic properties and soil microbial communities. Although soil in natural systems would have gotten progressively drier by July 2015 at lower elevation sites, agricultural production, such as almond farming, at locations near these elevations may continue year-round and promote favorable microbial growth conditions. During these particular California drought years, productive farmland had been fallowed from lack of water resources (Swain, 2015). Differences in the extent and type of activities at low and high elevations may be related to these observed diversity patterns, as emissions from the San Joaquin Valley contributed to deposited dust in response to human-induced disturbance at low elevations. Our isotopic analyses showed that dust deposited at these two low-elevation sites was predominantly from the San Joaquin Valley (Aciego et al., 2017), suggesting that a diverse suite of dust microorganisms may have been emitted from putatively parched soils early in the dry season during this record-breaking drought in California (Swain et al., 2014; Swain, 2015).

Our findings on dust variation across months and between years underscore the importance of drought and soil disturbances in driving microbial dispersal in montane ecosystems, including droughts in source locations. The extreme drought in California during both years of our study likely reduced vegetative cover and decreased soil stability (Field et al., 2010), which led to greater regional dust promotion. Locations like the San Joaquin Valley of California, characterized by heightened human activities, agricultural production, and soil disturbance, are particularly vulnerable to erosion during drought conditions and can emit large quantities of dust. Dust contributions to the Sierra Nevada from the San Joaquin Valley grew substantially between summer and fall 2014 (Aciego et al., 2017), suggesting that the microorganisms and nutrients in San Joaquin Valley dust may have played an increasingly important role in these ecosystems throughout the dry season. Although dust-associated microbial diversity was unchanged during this period, both fungal and bacterial composition differed monthly across each sampling year, providing partial support for our hypothesis that dust-associated microbial communities would vary throughout the dry season. In addition, as the percentage of dust inputs from Asia increased throughout the 2015 dry season during an extreme drought in Asia (Aarons et al., 2019), microbial community composition shifted by sampling month as well as by dust provenance. In comparison to the emerging 2015 drought in Asia, these California landscapes were already burdened by heightened dust emissions, and exacerbated by a multi-year drought coupled with regional land use practices. Likewise, these introduced Asian inputs yielded more heterogeneous dust mixtures deposited in the Sierra Nevada as the season became progressively drier.

Given the variation in Asian dust inputs as the 2015 drought emerged, we observed interannual variation in dust fungi deposited to the Sierra Nevada during two California drought years. In addition to compositional differences in fungal decomposers and mutualists, the relative abundance of dust-associated fungal pathogens differed across sampling years. Overall, these findings provided partial support for our hypothesis that source region droughts would drive interannual differences in dust microbial communities and fungal functional group abundance within this elevation gradient.

### Landscape Topography and Microbial Processes

Our finding that clinal increases in dust P concentrations spiked during our second sampling year suggests that this dust may have exerted ecosystem-level influences at high elevations. Previous studies have illustrated greater airborne flux of bioavailable P delivered to high elevation sites, with implications for terrestrial ecosystem productivity (O’Day et al., 2020). In this slowly eroding, dust-reliant montane ecosystem, dust-associated mineral P contributions elicit transformative effects on vegetation communities, soil and ecosystem development, and biogeochemical cycling (Arvin et al., 2017; Gu et al., 2019). As Longo et al. (2014) demonstrated in Mediterranean aerosols, a portion of this soluble organic P may be microbially derived. While little is known about how dust elemental concentrations affect microbial activity or drive the structure of dust-associated microbiomes in airborne ecosystems, some microorganisms may use airborne dust as refugia and access labile carbon compounds to fuel their metabolic activities.

Previous research in soil and subalpine snow shows that bacterial communities may be structured by environmental changes that increase clinally or co-vary with elevation (Bowers et al., 2012; Carey et al., 2016). Likewise, our results indicate that low and high elevational extremes may serve as environmental filters (Meier et al., 2010; Bahram et al., 2012; Zimmerman and Vitousek, 2012; Coince et al., 2014). However, although dust-associated microbial diversity and community composition differed by elevation, these patterns did not exhibit a clinal trend (i.e., changes were not proportional along the elevation gradient). Dust compositional and diversity changes by elevation may have been driven by a mixture of incoming microorganisms and ambient microbial communities at each site. Landscape features and upper canopy plants, such as conifers that host endophytic and phyllosphere microbiomes (Kinkel, 1991; Osono, 2006; Sieber, 2007; Frank et al., 2017), may release dust and microorganisms that then intermix with the aeolian microbiome. Abiotic factors like temperature and precipitation vary clinally along our elevation gradient, and the combination of these parameters shapes the realized niche (Vetaas, 2002) and corresponds with transition zones for dominant plants in our study locations.

We found that the diversity of plant-associated mycorrhizal fungal taxa increased with elevation, which may have corresponded with the compositional, biogeographic, or phenological patterns associated with plant hosts and differing environmental conditions at the highest elevations. In particular, a greater proportion of the fungal community at our highest elevation sites was composed of mutualistic fungal symbionts, such as lichen-forming fungi *Buellia griseovirens* and *Fulgidea sierrae. Fulgidea sierrae* has previously been identified from coniferous forests in montane ecosystems in California (Bendiksby and Timdal, 2013) and may have therefore originated from local sources and subsequently been entrained in Sierra Nevada dust. Our previous study from soil in a tropical montane cloud forest (TMCF, Looby et al., 2016) shows that the relative abundance of arbuscular mycorrhizal fungi decreases with increasing elevation during the dry season, while ectomycorrhizal fungal diversity increases with elevation during the TMCF wet season. Likewise, mutualistic fungi increased with elevation in our study’s temperate montane ecosystem, which may also correlate with increasing abundance or variety of ectomycorrhizal host trees at higher elevations. In contrast, *Trichophaea*, an ectomycorrhizal fungus from the Pyronemataceae family, was indicative of our lowest elevation site. This taxon belongs to a fungal group that emerges following disturbance, such as post-catastrophic wildfire (Glassman et al., 2016), so greater disturbances or warmer-drier conditions at this site may correspond to the abundance of this taxon.

Beyond mutualistic or host associations, indicators of higher elevation sites included several types of free-living basidiomycetous yeasts, including *Rhodotorula mucilaginosa, Vishniacozyma* spp., *Cryptococcus* spp., and *Naganishia* spp. Tremellomycetes were only indicators of the highest elevation sites; in particular, *Vishniacozyma* and *Naganishia* spp. contains taxa that are psychrophilic and have been isolated in extremely cold locations, such as from retreating glaciers in the Canadian High Arctic (Tsuji et al., 2019) and extreme high elevations in the Atacama region (Vimercati et al., 2016; Schmidt et al., 2017). Snowpack and colder temperatures at our highest elevation site likely promoted favorable conditions for cold-tolerant (i.e., psychrophilic) microbial taxa more than the warmer-drier conditions at our lower elevation sites. Either these taxa were equipped with traits to survive harsh atmospheric conditions during long-distance transport or the presence of these psychrophilic taxa may be an indication of ambient microbial inputs from depositional sites. Another indicator of our highest elevation sites was *Phlebia centrifuga*, a wood-decaying basidiomycete fungus from the Polyporales order, which has previously been touted as an indicator of old-growth forests in Nordic countries (Makela et al., 2018). *Phlebia centrifuga* has an exposed spore-bearing surface (hymenophore) and crust-like corticoid growth on decomposing tree trunks, often found in more pristine or unmanaged forests, and this orientation may have contributed to the abundance of basidiospores entering the dust microbiome at the highest elevation site.

### Intra-Seasonal Temporal Variation

We detected interannual temporal variation in both decomposer and mutualistic fungal communities. Only fungal mutualists exhibited monthly variation in community structure; these patterns persisted within years or for individual elevations. Fungi from the Arthoniales order also increased in abundance throughout the 2015 dry season. Members of this order are known to disperse long distances. Indeed, many dimorphic Arthonialean lichens reproduce both asexually, with soredia for airborne dispersal, and sexually, with ascospores (Tehler et al., 2009). Although this study did not sample microorganisms while in transit, tropical or subtropical Arthoniales taxa may have traveled along the jet stream to California early in the dry season but then potentially tapered off as the season progressed. In contrast, our analyses suggest that members of the Cystobasidiomycetes class were more abundant in the late 2015 dry season. Previous studies have shown that basidiomycete yeasts from Cystobasidiomycetes disperse together with lichen soredia as airborne contaminants traveling with lichen mycobionts (Cernajova and Skaloud, 2020). Although we did find an abundance of cold-adapted basidiomycete yeasts from Cystobasidiomycetes in higher-elevation dust samples, this did not correspond with abundance of lichen-forming fungi (Turchetti et al., 2013).

Consistent with our finding that dust from higher elevation sites harbored cold-adapted yeasts, the features of lower elevation sites may exert selective pressure to encourage taxa with traits facilitating their survival in warmer or drier conditions. For instance, a particularly abundant bacterial phylum at low-elevation sites, Firmicutes, consists of desiccation-resistant spore formers that may thrive in the hot-dry conditions found at these sites. Taxa from the phylum Acidobacteria were more abundant earlier than later in the 2015 dry season; this trend was likely driven by the abundance of Acidobacterial taxa early in the dry season at the Soaproot site. Previous studies have shown a remarkable rise in the relative abundance of Acidobacteria during dust events (Tang et al., 2018), specifically within the atmospheric environment of the Gobi Desert (Maki et al., 2017). We may have therefore expected rising Acidobacterial taxa richness or abundance as the 2015 dry season progressed, and more Asian dust entered our study system. Nevertheless, dust storm events in Asian deserts in 2015 likely contributed substantial Acidobacterial taxa to the atmosphere and subsequent deposition zones throughout the dry season.

### Human and Ecosystem Implications

Our findings on both temporal and elevation-linked differences in fungal functional group composition have important implications for recipient ecosystems and human health. In particular, the abundance and type of fungal pathogens present could determine the extent to which host taxa are infected and detrimentally affected by pathogenesis. Some airborne plant pathogens, such as those causing powdery mildews and sooty molds, may lead to crop losses or forest blights (Peetz et al., 2007; Carisse et al., 2008). Previous studies have shown that air pollution, which may be more prevalent at lower elevations, interacts with fungal pathogens to exacerbate asthmatic responses and leads to chronic pulmonary disease or organic toxic dust syndrome in human hosts (Rask-Andersen and Malmberg, 1990; Tunnicliffe et al., 2001; Peng et al., 2019). Overall, the persistence of these fungal groups likely has important consequences for ecological and human health.

Although our sequencing approach and molecular analyses may impart biases that constrain our interpretation of the total abundance of pathogenic reads, the effects of month and elevation on fungal pathogens provides partial support for our hypothesis that more dust-associated pathogens would be found lower in elevation. In our study, we detected the greatest relative abundance of fungal pathogenic sequences at lower elevations, and this pathogenic signal diminished at the highest elevation. There may be a greater dominance of fungal pathogens at the lowest elevation sites corresponding with more human activities and soil disturbance, as is characteristic of the San Joaquin Valley of California. The impact of elevation and sampling month on fungal pathogen richness, diversity, and the relative abundance of fungal pathogens suggests that interacting spatial and temporal factors may determine the spread and distribution of ecologically relevant pathogens.

Interactions among soil microbial communities at our sites and the incoming aeolian microbiome may yield unique compositional and functional attributes of the resultant dust-associated microbiome. Incoming microorganisms traveling long distances may be either active in the air (Dimmick et al., 1979; Tang et al., 2018) or dormant such that they maintain viability upon deposition (Prospero et al., 2005). To stay viable, airborne microorganisms must be equipped with traits that allow them to withstand turbulent conditions and long-distance transport (Chagnon et al., 2013; Krause et al., 2014; Singh et al., 2019). Conditions at recipient sites may determine whether deposited microorganisms perish, proliferate, or associate with plant, animal, or human hosts (Schmidt et al., 2020).

The elevation-related shifts we observed in plant-associated mycorrhizal fungi likely have functional implications for resident biotic communities and contribute to patterns in ecosystem function across this elevational gradient. In general, the functional capacity of fungal communities can have transformative effects on ecosystems, as both decomposer and mutualistic fungi alter decomposition rates and are the engines of nutrient cycling, including carbon and macro-nutrient cycles (Ehrenfeld et al., 2005), which may be particularly important in slowly eroding montane systems.

### Study Limitations and Future Research

Although our study is one of the first to investigate how microbial communities and the chemical composition of dust change along an elevational gradient, it did have some limitations. For instance, our 16S rRNA gene analyses of bacterial communities yielded many sequences similar to eukaryotic photosynthetic taxa, which may have been better examined with sequencing technologies targeting longer genomic portions, such as single-molecule real-time sequencing using the PacBio Sequel Systems (Menlo Park, CA, United States) platform. Although previous studies have reported complex species-specific interactions between bacteria and microalgae, this phenomenon remains understudied (Schwenk et al., 2014). As microalgae can survive the harsh conditions of airborne transport, soil or dust-associated microorganisms have likewise evolved photoprotective pigments to survive environmental stress (Rehakova et al., 2019). However, the activity of airborne bacteria across varying temperatures and their taxon-specific interactions with photosynthetic micro-eukaryotes would both be promising avenues for future aeolian microbial research, especially throughout the dry season in California (Maki and Willoughby, 1978).

Because of the remoteness of our sampling locations and constraints associated with our sampling campaigns, dust collectors were deployed for varying durations of time prior to collection. Although the durations differed for dust collections across samples, deployment duration and dust mass was unrelated to microbial diversity or richness, ostensibly because dust flux is variable on annual timescales. After deposited dust accumulated, we subsequently extracted DNA from passive dust collectors by suspending this dust into sterile water and transporting it before performing DNA extraction and molecular analyses. While collectors were sterilized before placement each year, we subsequently replaced collectors after rinsing only with sterile water, such that relic DNA may have remained in our replaced collectors following rinsing. This may have confounded our interpretation of temporal variation in microbial community structure or chemical analyses of dust within a given sampling year, although it would not have impacted elevation-based findings. Our methods were also unable to discern between active microorganisms and dead microbial fragments or relic DNA that may have accumulated with our collected dust. However, we repeated all community composition and diversity analyses with binary data indicating presence/absence of individual taxa and taxa richness, which revealed that trends identified based on relative abundance were also identified in the presence/absence data. Therefore, we are confident in our reporting and interpretation of dust-associated microbial communities over time.

In addition, our molecular and bioinformatic techniques were not able to differentiate between viable DNA and extracellular DNA from dead microorganisms, which may persist for several years and obfuscate estimates of extant microbial diversity (Carini et al., 2017). Without quantifying relative ribosomal quantities (Placella et al., 2012), using stable isotope probing techniques to calculate excess atom fractions (Hungate et al., 2015), applying transcriptional and (Placella and Firestone, 2013) metatranscriptional techniques (Shakya et al., 2019), or measuring CO_2_ pulses and microbial metabolism in dust (upon deposition or with exposure to favorable conditions), it would be challenging to discern the active portion from relic molecular products. Although our study was not designed to tease apart the relative contributions of global vs. local microbial inputs to the dust microbiome, higher intercontinental transport may have resulted in more exogenous microbial deposits. Yet, since both bacterial and fungal communities were correlated with the radiogenic nuclides used to determine dust provenance, microorganisms traveling long distances may have experienced selective forces during long distance transit.

Another avenue for future study would be to analyze dust and soil physicochemical properties, as well as soil and phyllosphere microbial communities at these study sites. Although no dust samples were analyzed for pH, we acknowledge that dust pH or ambient moisture measurements could have aided in the interpretation of our results, as certain microorganisms have specific tolerance ranges. Removing leaf cuticles or the use of specialized instrumentation, such as an ultrasonic bath, to separate surficial leaf associations from endophytic microorganisms could provide insight into ambient microbial inputs from local sites (Guzman et al., 2020). Analyzing microbial communities from transoceanic dust source locations, such as the Gobi or Taklamakan Deserts (Aciego et al., 2017), would provide valuable baseline data for bioinformatically teasing apart local vs. long-distance contributions to the dust-associated microbiome. Weather station instrumentation at source and recipient locations, coupled with soil biogeochemical measurements across locations, may also help quantify the relative impact of incoming dust on ecosystem function. Although soil biogeochemistry was beyond the scope of this study, our future research will examine both dust- and soil-based microbial communities and soil biogeochemical cycling across unusually dry years and an unusually wet year along this same elevation gradient.

## Conclusion

Our results suggest that landscape topography and drought events in dust source locations may alter the composition and diversity of dust-associated microorganisms deposited into a temperate montane ecosystem. Each year of our study, we found that elevation determined dust-microbial community composition, suggesting elevation filtered the microbial inputs from dust and the ambient microorganisms at each site intermixed with incoming microorganisms to yield distinct structural and functional attributes. Dust-associated microorganisms play numerous functional roles in ecosystems, and dust biogeochemistry has implications for human health, biodiversity, and food security. Factors exacerbated by global change, including drought and development in dust source locations, may result in drier, unstable landscapes, increasing susceptibility to wind erosion and amplifying dust-promoting conditions in the future. Therefore, our findings have crucial implications for how dust-associated microbial dispersal may respond to future climate scenarios, providing critical information on dust-driven microbial dynamics and biogeography in montane ecosystems. These data provide evidence that elevation and time since the last precipitation event may influence the structure and functioning of dust-associated microbial communities. Our study underscores the importance of drought events in dust source locations on long-distance microbial dispersal and the interactions among temporal factors and landscape topography on dust-associated microbial communities in montane ecosystems.

## Materials and Methods

### Study Sites

Study sites were in California, United States, on the western side of the Sierra Nevada in the Southern Sierra Critical Zone Observatory (SSCZO). Sites ranged from oak savannah to subalpine forest, crossing the rain-snow transition zone (Supplementary Figure 1). These sites include the San Joaquin Experimental Range (SJER; 400 m above sea level), Soaproot (1,100 m), Providence (2,000 m), and Short Hair (2,700 m) (sites described in detail in O’Geen et al., 2018). The region is characterized by a Mediterranean climate with cool, wet winters and warm, dry summers with generally little to no precipitation from mid-May to October (i.e., dry season). From 2012 to 2015, the region experienced severe drought (Robeson, 2015).

The unprecedented magnitude of the drought in California during 2012–2015 (Griffin and Anchukaitis, 2014; Diffenbaugh et al., 2015; Robeson, 2015) resulted in low soil moisture, increasing the likelihood for wind erosion. This may be especially relevant in locations characterized by frequent soil disturbance from tillage, such as in California’s San Joaquin Valley. During 2015, northern China—and large areas of northeastern Asia—experienced their most severe drought in the past 60 years (Wang and He, 2015). According to the Standardized Precipitation Evapotranspiration Index-(SPEI database), large areas of Mongolia, north China and northeastern Asia were under heightened drought conditions in July–October 2015 (Vicente-Serrano et al., 2010).

Dust contributions from the San Joaquin Valley grew significantly throughout the dry season in 2014, suggesting that a significant portion of dust was attributable to the extreme regional drought in California (Aciego et al., 2017). In contrast, the percentage of dust inputs from Asia increased throughout the 2015 dry season (Aarons et al., 2019). We determined that these changes were likely related to the prolonged drought in Asia during our second year of sampling. This remarkable interannual pattern highlights both the sensitivity of dust production and transport to drought and the teleconnections of dust transport in terrestrial ecosystems.

### Field Dust Collection

We used passive dust collectors (Nordic Ware, St. Louis Park, MN, United States) to characterize dust-associated bacterial and fungal community composition during the 2014 and 2015 dry seasons along this ∼2,300 m elevational transect (Figure 1). Dust samples were collected between July and October 2014 and again between July and October 2015. Five replicate dust collectors were deployed per location. In 2014, we deployed dust collectors in response to worsening drought, with collectors deployed at SJER, Soaproot, and Providence sites between June 6–7, 2014, and at Short Hair on July 5, 2014. Later deployment at the Short Hair site was necessary due to snowpack, which prevented earlier access. Dust collectors were sampled from SJER, Soaproot, and Providence between July 5–6, 2014 and then redeployed. Collectors were then sampled from all four sites on August 6–7, Sept 6–7, and October 8–9, 2014, for deployment lengths of 29–31 days. Collectors were sampled for microbial communities in July, August, and October 2014, and were retained for isotopic and elemental analyses in July, August and September 2014 (see Aarons et al., 2019). In 2015, we maximized the duration of collection due to limited resources, deploying collectors at each site between April 2 and 4, 2015. We collected samples on July 15 and between October 7 and 10, 2015, resulting in a deployment length of 103–104 days for the first sample and 91–93 days for the second.

Constraints precluded us from obtaining isotopic analyses from October 2014; therefore, we used isotopic and other dust-analysis data from samples collected in September 2014, 29 days previously, as a proxy for October 2014, to complement our molecular analyses of dust collected during that sampling month. In July and August 2014, and July and October 2015, we paired molecular analyses with the results of our isotopic fingerprinting and trace elemental analyses (Aciego et al., 2017; Aarons et al., 2019) of collected dust.

Our passive collectors (as per Aciego et al., 2017), which consisted of Teflon-lined Bundt pans, secondarily lined with Kevlar mesh (Industrial Netting Inc., Maple Grove, MN, United States) and filled with glass marbles (12.7 mm diameter; Brooklyn, NY, United States; Figure 1), were used to collect dust at the four study sites. Prior to deployment in the field, all collection materials were acid washed in 2 M HCl, with rinses of 18.2 MΩ water between each reagent cleaning step, prior to deployment.

We deployed collectors 2 m above ground level atop wooden posts within open-canopy locations at each site. Collectors were deployed upon these posts to minimize the contribution of local particulate material from nearby canopy trees or surface soil saltation. To recover dust-microbial samples from the collectors in the field, we used 18.2 MΩ water to rinse the marbles within the collector and then transferred the water-and-dust suspension to acid-washed 1 L Nalgene bottles (low density polyethylene; Figure 1), which were subsequently processed back at the laboratory.

### Sample Processing for Elemental and Isotopic Analyses

Samples were frozen and processed using techniques previously described in Aciego et al. (2009); Aarons et al. (2019), and using procedures outlined in Koornneef et al. (2015). Frozen samples were melted and immediately filtered to exclude the soluble contribution with consecutive 30 and 0.2 μm polycarbonate filters. Following filtration, these samples were weighed for dust mass (weights, m * g) and then dust was dissolved directly off of the 0.2 μm filter using techniques previously described in Aciego et al. (2009). We calculated dust heterogeneity as detailed in Aarons et al. (2019), using the dust source location concentrations of Sr in San Joaquin Valley and Asian dust endmembers (Ingram and Lin, 2002; Yokoo et al., 2004) to calculate the proportion of San Joaquin Valley and Asian dust in each measured sample. We used endmember isotope-mixing models, as modified from White (2013), to determine the relative inputs of San Joaquin Valley-derived dust vs. Asian-derived dust in our dust mixtures: ^87^Sr/^86^Sr_M_ = [^87^Sr/^86^Sr_A_ * f * (Sr_A_/Sr_M_)] + [^87^Sr/^86^SrB * (1—f) * Sr_B_/Sr_M_)]. Since our dust mixtures varied by the proportion of Asian dust in the samples (Aciego et al., 2017; Aarons et al., 2019), we used the proportion of Asian dust as the metric of dust heterogeneity.

Aliquots of each sample were measured for P or S concentrations on a Nu Instruments Attom high-resolution inductively coupled plasma mass spectrometer at the University of California Irvine. These aliquots were diluted in 2% HNO_3_ and blanks, standards, and samples were spiked with a mixed internal standard prior to analyses, as per Aarons et al. (2019).

### Laboratory Dust Filtration

We filtered dust suspensions into an acid-wash sterilized glass funnel through sterile 0.2 μm filters (47-mm diameter; Pall Supor 200 Sterile Grid filters, Pall Corporation, Port Washington, NY, United States) into a collecting flask below the acid-wash sterilized vacuum filtration unit. Filters were then stored in sterile Whirlpak bags at –20°C. To test for contamination, we filtered 1 L sterile Milli-Q water through a blank 0.2 μm filter using the same filtration apparatus as a negative control, which was processed alongside the dust samples. After samples were extracted and amplified, blank filters were free of DNA, indicating that contamination did not occur during processing.

### Molecular Analyses

We extracted microbial DNA from frozen 0.2 μm filters (Pall, NY, United States) using a MO BIO PowerWater DNA Isolation Kit (MO BIO Laboratories, Carlsbad, CA, United States; Qiagen Inc.), followed by cell lysis directly from filters, following the manufacturer’s instructions. Our DNA extracts were then quantified using a NanoDrop 2000/2000c UV-Vis spectrophotometer (Thermo Fisher Scientific, Wilmington, DE, United States). For prokaryotes, we amplified DNA extracts in duplicate using primers targeting the V3-V4 region of the 16S rRNA gene (S-D-Bact-0341-b-S-17 and S-D-Bact-0785-a-A-21; Klindworth et al., 2013). We conducted polymerase chain reaction (PCR) by combining 2.5 μL DNA template, 5 μL each of 1 μM forward and reverse primers, and 12.5 μL KAPA HiFi HotStart ReadyMix (KAPA Biosystems, Wilmington, MA, United States), totaling a 25 μL reaction. Thermocycler conditions were as follows: 95°C for 3 min, followed by 25 cycles of 95°C for 30 s, 55°C for 30 s, 72°C for 30 s, followed by an extension step for 5 min at 72°C. After amplification, we combined and purified the duplicate PCR products using Agencourt AMPure XP Beads (Beckman Coulter Genomics, Danvers, MA, United States). For purified bacterial amplicons, we conducted a second round of PCR to attach dual indices and sequencing adapters to each amplified sample using the Nextera XT Index Kit (Illumina, San Diego, CA, United States). For each of these reactions, 5 μL DNA, 5 μL each of 1 μM forward and reverse index primers, 25 μL KAPA HiFi HotStart ReadyMix, and 10 μL PCR grade water were combined to create a 50 μL reaction. Thermocycler conditions were programmed as follows: 95°C for 3 min, followed by 8 cycles of 95°C for 30 s, 55°C for 30 s, 72°C for 30 s, followed by an extension step for 5 min at 72°C. We then conducted a second purification step using AMPure Beads (described above) on our indexed amplicons. Next, we quantified our purified PCR products using the Quant-iT PicoGreen dsDNA assay kit (Life Technologies, Grand Island, NY, United States). As a final step, we pooled samples in equimolar concentrations and sequenced them using an Illumina MiSeq instrument at the University of California Riverside (UCR) Genomics Core Facility.

To target fungal amplicons, we used modified versions of the universal fungal primers ITS1F and ITS2 (described in Smith and Peay, 2014), which were improved as part of the Earth Microbiome Project (Walters et al., 2016). This primer set accurately identifies fungi to the specific epithet level, but may have limited phylogenetic resolution (Schoch et al., 2012; Lindahl et al., 2013; Öpik et al., 2014; Yarza et al., 2017). We performed PCR amplification in 25 μL reactions including 1 μL of 10 μM for each primer (forward and reverse), 1 μL DNA, 12.5 μL of Taq 2X Master Mix (New England Biolabs, Ipswich, MA, United States) and 9.5 μL diH2O, as per Collins et al. (2016). Thermocyclers were programmed to 94°C for 3 min, followed by 35 cycles of 94°C for 45 s, 50°C for 60 s, and 72°C for 90 s, with a final extension step at 72°C for 10 min. As described in Walters et al. (2016), our forward primer sequences contained unique 12-base Golay barcodes as indexes for this single indexed reaction (Hamady et al., 2008). For fungal amplicons, we purified our PCR products using a NucleoSpin Gel-Extraction kit (Macherey-Nagel GmbH &Co. KG, Düren, Germany). Purified samples were quantified with a NanoDrop 2000 (Thermo Fisher Scientific, Wilmington, DE, United States), prior to pooling in equimolar concentrations. We sequenced our library of fungal amplicons in a multiplexed run on the Illumina MiSeq platform (Illumina Inc., San Diego, CA, United States) at UCR Genomics Core Facility. A total of 2,490,644 paired-end sequence reads were deposited to the Sequences Read Archive (SRA) database associated with BioProject accession number SUB10961273; with 7,548,435 paired end sequences submitted databases associated with BioSample submission accession SUB1096275 Southern Sierra Dust Targeted loci environmental.

### Bioinformatics

Fungal ITS1 amplicon sequences were analyzed with AMPtk: the Amplicon Toolkit for NGS data (formerly UFITS; v1.2.4; Palmer et al., 2018), as per Pombubpa et al. (2020). Demultiplexed paired-end sequences were pre-processed, respectively, as both forward and reverse reads, trimmed to a maximum of 300 bp; any read with lengths below 100 bp in length was discarded. Quantitative Insights into Microbial Ecology (QIIME; Kuczynski et al., 2012) was used to quality filter 16S amplicon sequences and determine taxonomic identity against the SILVA reference database (Quast et al., 2013), using 97% similarity for OTUs. Singletons were removed and analysis of similarity was performed in QIIME for 16S rRNA genes.

### Functional or Microbial Group Analyses

We explored the significance of dust-associated phylogenetic taxa detected from libraries constructed for 16S rRNA or ITS1 targeted amplicon sequencing. To examine responses of the fungal community (ITS1), we assigned OTUs to functional groups using the online application FUNguild “http://www.stbates.org/guilds/app.php” 2019-02-12, Nguyen et al., 2016). After processing OTUs through FUNguild, our OTUs were assigned to Taxon, Trophic Mode, Guild, and Growth Morphology. FUNguild functional group assignments were as follows: pathotrophs, pathotroph-saprotrophs and pathotroph-symbiotrophs were assigned to the “pathogen group,” saprotrophs and saprotroph-pathotrophs were assigned to the “decomposer group,” and all saprotroph-symbiotrophs and symbiotrophs were assigned to the “mutualist group.” We kept only FUNguild assignments at the confidence level of “highly probable (Supplementary Table 3),” removing all taxa that were at the confidence level of “possible” from the analyses. Finally, we compared the relative abundance of prokaryotic phyla present above a 1% threshold across conditions in the study.

### Statistical Analyses

We used the “diversity” function in the “vegan” package to calculate Shannon’s Diversity (i.e., alpha diversity) with the Shannon-Wiener index, using rarefied OTU counts. We used the “rrarefy” function from the “vegan” package, as per Oksanen et al. (2012), with bacterial counts rarefied to 34,559 or fungal counts rarefied to 7,302. After singleton sequences were removed, raw OTU counts were used to calculate taxa richness as to not lose rare taxa that could be present. We reported adjusted *p*-values that were calculated using the Bonferroni correction with the “adjust_*p*-value” function from the “rstatix” package in R to control the family-wise error rate. For linear regressions, adjusted *R*^2^ values were calculated using the “stat_regline_equation” function within the “ggpubr” package in R (Kassambara, 2020).

We fit linear models using the “lm” function from the “stats” package in R, with bacterial alpha diversity, bacterial taxa richness, fungal alpha diversity, or fungal taxa richness as response variables and elevation, concentrations of radiogenic isotopic composition, concentrations of trace elements, month, or year as predictive variables. ANOVA and Tukey’s Honest Significant Differences *post-hoc* tests were used to determine if there were significant differences between elevations or sampling timepoint as categorical variables. We used the “aov” function to compare the variation in these response variables within and among sites, sampling months and sampling years, or time in the dry season. To evaluate whether elevation or sampling timepoint affected microbial or functional group diversity, richness, or relative abundance, we used the “kruskal.test” function from the “stats” package; if significant, we tested the significance of pairwise comparisons. To control for the family-wise error rate, Bonferroni corrections were applied to all reported statistical tests using the “adjust *p*-value” function from the “rstatix” package (Kassambara, 2021). All statistical analyses were performed in R version 4.0.5 (R Core Team, 2018).

Beta diversity and community composition were calculated and visualized for each locus using principal coordinate analyses (PCoA) of the associated relativized feature table (McMurdie and Holmes, 2013; Borcard et al., 2018). We calculated distance matrices using the Bray–Curtis Dissimilarity Index from relativized OTU counts using the “decostand” (method = total) function from “vegan.” We standardized varying sequence depths with the “total” option in the “decostand” function in the “vegan” package in R, transforming raw counts into proportions of each OTU per sample. With the “betadisper” function, we checked for between-group dispersion in fungal or bacterial composition between early or late timepoints within the dry season. For dust heterogeneity, we performed linear regressions using microbial alpha diversity (calculated from rarefied data) or taxa richness for fungi and bacteria. We also used dust heterogeneity to produce ordinations for visualizing compositional differences across microbial groups.

As dispersion in fungal and bacterial composition did not significantly differ by early vs. late dry season sampling timepoints, we decided to conduct permutational multivariate analysis of variance (PERMANOVA) with the “vegan” function “adonis” and “adonis2” in R (999 permutations; Oksanen et al., 2012) with relativized Bray–Curtis dissimilarity matrices to check between group differences and evaluate how composition varied across groups.

Using PERMANOVA, we assessed significant differences in composition between sampling years, sampling months, or abiotic parameters—such as the concentration of radiogenic isotopic composition, concentration of trace metals, or macronutrients—on compositional changes. Site was used as a blocking variable (strata) to restrict permutations within a particular site, as appropriate. Beta diversity and community composition were visualized for each locus using principal coordinate analyses (PCoA) of the associated relativized feature table. Additionally, we relativized by phyla and class, respectively, for bacteria and fungi and conducted PERMANOVAs to compare the composition of fungal and bacterial classes, phyla, as well as by fungal functional groups, by site, month, year, or time in the dry season.

For Canonical Correspondence Analysis (CCA), we ran a Detrended Correspondence Analysis (DCA) on the relativized feature tables for fungi and bacteria, respectively, using the “decorana” function from the “vegan” package to determine the homogeneity and respective variation in these data. Concentrations of trace elements and radiogenic isotopic composition were scaled prior to running the DCAs and CCAs using the “scale” function from “R-base” package. Based on the length of the first DCA axis, we ran CCAs using the relativized feature table and all associated metadata. We calculated adjusted *R*^2^ value to obtain the overall variation explained by the CCA models. Then, ANOVAs were run on the CCA model to determine which environmental variables contributed most to the variation within the composition data (using RsquareAdj and anova from the “vegan” package). Finally, we used the “ordistep” function from the “vegan” package to determine which environmental variables significantly constrained the variation in the composition data. Only significant environmental drivers were shown in CCA plots.

We used “decostand,” to examine the relationship between the relative abundance of the most abundant bacterial or fungal classes by elevation and sampling month, across both years of the study (Supplementary Figure 5). We used non-parametric Kruskal-Wallis rank sum tests when the response variable was not normally distributed. Also using “decostand,” we calculated the relative abundance of FUNGuild-assigned taxa (Nguyen et al., 2016), binned into fungal functional groups. We fit linear models using the “lm” function from the “stats” package in R to compare the richness or diversity of functional or taxonomic groups by elevation, month, or year, as well as to examine the relationship between the relative abundance of pathogenic, mutualistic, or decomposer fungi across the elevation gradient. We used dust heterogeneity, as the ranked proportion of Asian dust in the dust mixtures, to produce ordinations for visualizing compositional differences across microbial groups. Additionally, we constructed linear models to investigate interacting effects of elevation and sampling month, or month within year, on the richness, diversity, and relative abundance of these fungal groups. Adjusted *R*^2^ values for these regressions were calculated using the “stat_regline_equation” function from the “ggpubr” package (Kassambara, 2020).

### Indicator Species Analysis

We tested for indicator taxa associated with each elevation for the ITS1 region, with a particular interest in fungal taxa at the highest or lowest elevations. We calculated indicator values using the “multiplatt” function with 9,999 permutations in the “indicspecies” R package (De Caceres and Legendre, 2009). Indicator value indices are used for assessing the predictive value of taxa as indicators of conditions present in the different groups (De Caceres and Legendre, 2009). We only retained taxa with a *p*-value < 0.05 as significant (Supplementary Table 3).

## Data Availability Statement

The datasets presented in this study can be found in online repositories. The names of the repository/repositories and accession number(s) can be found below: https://ncbi.nlm.nih.gov/bioproject/PRJNA797677, NCBI Sequence Read Archive—PRJNA797677.

## Author Contributions

SH, EA, CC, SAa, SAc, and JB conceived of the study. SH, EA, CC, JB, ND, MBl, MM, NP, SAc, SAa, and MBa executed the study, collected the samples, processed samples, and generated data. EA, SH, JS, SAa, and SAc provided supplies, instrumentation, and funding to conduct analyses. HF, NP, and MM analyzed data and generated figures. MM wrote the manuscript. SH, HF, EA, CC, ND, MBl, MBa, MM, NP, SAa, and SAc edited the manuscript and commented on description of the study. All authors contributed to the article and approved the submitted version.

## Conflict of Interest

The authors declare that the research was conducted in the absence of any commercial or financial relationships that could be construed as a potential conflict of interest.

## Publisher’s Note

All claims expressed in this article are solely those of the authors and do not necessarily represent those of their affiliated organizations, or those of the publisher, the editors and the reviewers. Any product that may be evaluated in this article, or claim that may be made by its manufacturer, is not guaranteed or endorsed by the publisher.

## Supplementary Material

Supplementary Figure 1 Bacterial 16S rRNA marker gene alpha diversity **(A)** and fungal ITS1 region alpha diversity **(B)** in dust collected during either 2014 or 2015 dry seasons in this study.

Supplementary Figure 2 Bacterial 16S rRNA marker gene alpha diversity **(A)** and fungal ITS region alpha diversity **(B)** across elevations during both years of the study.

Supplementary Figure 3 The relative abundance of 16S rRNA gene bacterial phyla in 2014 **(A)** and 2015 **(B)** and the relative abundance in ITS1 region of fungal classes in 2014 **(C)** and 2015 **(D)** across elevations.

Supplementary Figure 4 The relative abundance of microbial groups detected through 16S rRNA marker gene amplification targeting bacterial 16S rRNA gene and fungal ITS1region amplicons across the dry season. The relative abundance of Acidobacteria **(A)** and Cyanobacteria **(B)** and Arthoniomycetes **(C)** were more abundant early in the dry season than later that season; the relative abundance of Cystobasidiomycetes **(D)** increased later in the dry season. Fungal ITS1 taxa summaries are shown by elevation **(E)**.

Supplementary Table 1 Site and sampling characteristics. Coordinates, elevation, and deployment dates for each of four sites in the elevation gradient in the Sierra Nevada of California. The International Geo Sample Number (IGSN) is an alphanumeric code that uniquely identifies samples taken from the environment.

Supplementary Table 2 Fungal taxa representing fungal tropic modes. Fungal taxa associated with each functional group, as assigned through FUNguild (as per Nguyen et al., 2016).

Supplementary Table 3 Indicator species analyses for significant fungal taxa (to genus- or species-level). Only significant p-values (*p* < 0.05) reported.
