# Supplementary figures and images for: Landscape Topography and Regional Drought Alters Dust Microbiomes in the Sierra Nevada of California
Source: Front Microbiol. 2022 Jun 28;13:856454. doi: 10.3389/fmicb.2022.856454 (PMC9274194; doi:10.3389/fmicb.2022.856454)

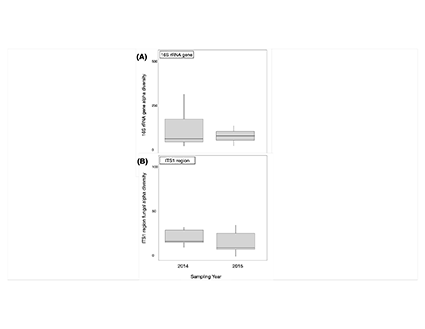

Supplement: Supplementary Figure 1 — Bacterial 16S rRNA marker gene alpha diversity (A) and fungal ITS1 region alpha diversity (B) in dust collected during either 2014 or 2015 dry seasons in this study. [file Image_1.TIFF]

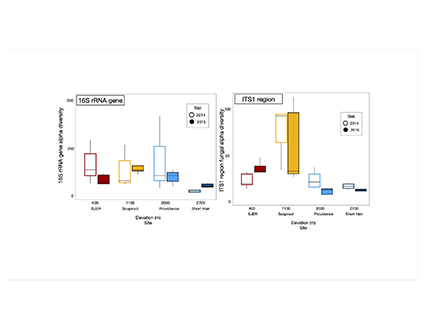

Supplement: Supplementary Figure 2 — Bacterial 16S rRNA marker gene alpha diversity (A) and fungal ITS region alpha diversity (B) across elevations during both years of the study. [file Image_2.TIFF]

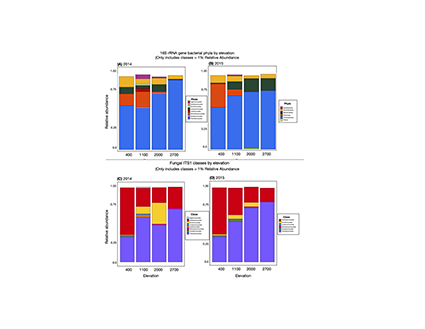

Supplement: Supplementary Figure 3 — The relative abundance of 16S rRNA gene bacterial phyla in 2014 (A) and 2015 (B) and the relative abundance in ITS1 region of fungal classes in 2014 (C) and 2015 (D) across elevations. [file Image_3.tiff]

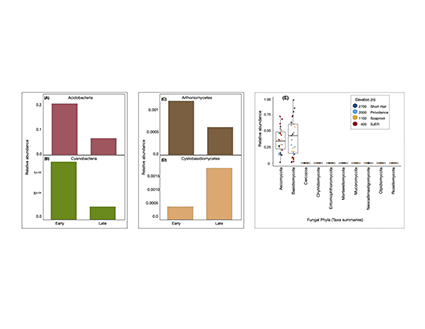

Supplement: Supplementary Figure 4 — The relative abundance of microbial groups detected through 16S rRNA marker gene amplification targeting bacterial 16S rRNA gene and fungal ITS1region amplicons across the dry season. The relative abundance of Acidobacteria (A) and Cyanobacteria (B) and Arthoniomycetes (C) were more abundant early in the dry season than later that season; the relative abundance of Cystobasidiomycetes (D) increased later in the dry season. Fungal ITS1 taxa summaries are shown by elevation (E). [file Image_4.tiff]
