# Supplementary material for: Landscape Topography and Regional Drought Alters Dust Microbiomes in the Sierra Nevada of California
Source: Front Microbiol. 2022 Jun 28;13:856454. doi: 10.3389/fmicb.2022.856454 (PMC9274194; doi:10.3389/fmicb.2022.856454)
Supplement: Supplementary Table 1 — Site and sampling characteristics. Coordinates, elevation, and deployment dates for each of four sites in the elevation gradient in the Sierra Nevada of California. The International Geo Sample Number (IGSN) is an alphanumeric code that uniquely identifies samples taken from the environment. [file Table_1.docx]

| Site | IGSN | Sample ID | Latitude (ºN) | Longitude (ºW) | Elevation (m) | Dust sampling dates | Deployment (days) |
| --- | --- | --- | --- | --- | --- | --- | --- |
| SJER | IE117005 | JJ114 | 37.10794 | 119.73198 | 400 | 06/07/14-07/05/14 | 30 |
| Soaproot | IE117007 | JT314 | 37.03053 | 119.25817 | 1,000 | 06/07/14-07/05/14 | 30 |
| Providence | IE117001 | JP414 | 37.06008 | 119.18268 | 2,000 | 06/06/14-07/05/14 | 31 |
| SJER | IE117005 | AJ414 | 37.10794 | 119.73198 | 400 | 07/06/14-08/06/14 | 33 |
| Soaproot | IE117007 | AT414 | 37.03053 | 119.25817 | 1,000 | 07/06/14-08/06/14 | 33 |
| Providence | IE117001 | AP114 | 37.06008 | 119.18268 | 2,000 | 07/05/14-08/07/14 | 33 |
| Short Hair | IE117003 | AS114 | 37.06698 | 119.98711 | 2,700 | 07/05/14-08/07/14 | 33 |
| SJER | IE117005 | SJ514 | 37.10794 | 119.73198 | 400 | 08/06/14-09/06/14 | 31 |
| Soaproot | IE117007 | ST414 | 37.03053 | 119.25817 | 1,000 | 08/06/14-09/07/14 | 32 |
| Providence | IE117001 | SP614 | 37.06008 | 119.18268 | 2,000 | 08/07/14-09/07/14 | 31 |
| Short Hair | IE117003 | SS214 | 37.06698 | 119.98711 | 2,700 | 08/07/14-09/06/14 | 30 |
| SJER | IE117005 | OJ514 | 37.10794 | 119.73198 | 400 | 09/06/14-10/08/14 | 32 |
| Soaproot | IE117007 | OT414 | 37.03053 | 119.25817 | 1,000 | 09/06/14-10/8/14 | 32 |
| Providence | IE117001 | OP614 | 37.06008 | 119.18268 | 2,000 | 09/07/14-10/09/14 | 32 |
| Short Hair | IE117003 | OS214 | 37.06698 | 119.98711 | 2,700 | 09/06/14-10/08/14 | 33 |
| SJER | IE117005 | JJ415 | 37.10794 | 119.73198 | 400 | 04/04/15-7/15/15 | 102 |
| SJER | IE117005 | JJ515 | 37.10794 | 119.73198 | 400 | 04/04/15-7/15/15 | 102 |
| Soaproot | IE117007 | JT115 | 37.03053 | 119.25817 | 1,000 | 04/03/15-7/15/15 | 101 |
| Soaproot | IE117007 | JT415 | 37.03053 | 119.25817 | 1,000 | 04/03/15-7/15/15 | 101 |
| Providence | IE117001 | JP315 | 37.06008 | 119.18268 | 2,000 | 04/04/15-07/15/15 | 100 |
| Providence | IE117001 | JP515 | 37.06008 | 119.18268 | 2,000 | 04/04/15-07/15/15 | 100 |
| Short Hair | IE117003 | JS115 | 37.06698 | 119.98711 | 2,700 | 04/02/15-07/15/15 | 104 |
| Short Hair | IE117003 | JS415 | 37.06698 | 119.98711 | 2,700 | 04/02/15-07/15/15 | 104 |
| SJER | IE117005 | OJ415 | 37.10794 | 119.73198 | 400 | 07/15/15-10/07/15 | 91 |
| SJER | IE117005 | OJ515 | 37.10794 | 119.73198 | 400 | 07/15/15-10/07/15 | 91 |
| SJER | IE117005 | OJ615 | 37.10794 | 119.73198 | 400 | 07/15/15-10/07/15 | 91 |
| Soaproot | IE117007 | OT115 | 37.03053 | 119.25817 | 1,000 | 07/15/15-10/08/15 | 92 |
| Soaproot | IE117007 | OT415 | 37.03053 | 119.25817 | 1,000 | 07/15/15-10/08/15 | 92 |
| Soaproot | IE117007 | OT515 | 37.03053 | 119.25817 | 1,000 | 07/15/15-10/08/15 | 92 |
| Providence | IE117001 | OP315 | 37.06008 | 119.18268 | 2,000 | 07/15/15-10/10/15 | 94 |
| Providence | IE117001 | OP515 | 37.06008 | 119.18268 | 2,000 | 07/15/15-10/10/15 | 94 |
| Providence | IE117003 | OP615 | 37.06008 | 119.18268 | 2,000 | 07/15/15-10/10/15 | 94 |
| Short Hair | IE117003 | OS115 | 37.06698 | 119.98711 | 2,700 | 07/15/15-10/09/15 | 93 |
| Short Hair | IE117003 | OS415 | 37.06698 | 119.98711 | 2,700 | 07/15/15-10/09/15 | 93 |
| Short Hair | IE117003 | OS615 | 37.06698 | 119.98711 | 2,700 | 07/15/15-10/09/15 | 93 |
