# Supplementary material for: Landscape Topography and Regional Drought Alters Dust Microbiomes in the Sierra Nevada of California
Source: Front Microbiol. 2022 Jun 28;13:856454. doi: 10.3389/fmicb.2022.856454 (PMC9274194; doi:10.3389/fmicb.2022.856454)
Supplement: Supplementary Table 2 — Fungal taxa representing fungal tropic modes. Fungal taxa associated with each functional group, as assigned through FUNguild (as per Nguyen et al., 2016). [file Table_2.docx]

**Supplemental Table 2. Fungal taxa representing fungal tropic modes.**

Fungal taxa associated with each functional group, as assigned through FUNguild (as per Nguyen *et al*., 2016).

| Taxon | Trophic Mode |
| --- | --- |
| *Cystobasidium pinicola* | Pathotroph |
| *Cystobasidium slooffiae* | Pathotroph |
| *Cystobasidium psychroaquaticum* | Pathotroph |
| *Cystobasidium* | Pathotroph |
| *Collophora* | Pathotroph |
| *Cosmospora viridescens* | Pathotroph |
| *Didymocyrtis cladoniicola* | Pathotroph |
| *Pseudocercospora* | Pathotroph |
| *Thyrostroma* | Pathotroph |
| *Beauveria* | Pathotroph |
| *Myrangium* | Pathotroph |
| *Ascosphaera* | Pathotroph |
| *Taphrina* | Pathotroph |
| *Aplosporella* | Pathotroph |
| *Teratosphaeriaceae* | Pathotroph-Saprotroph |
| *Stemphylium* | Pathotroph-Saprotroph |
| *Coniothyrium* | Pathotroph-Saprotroph |
| *Monodictys* | Pathotroph-Saprotroph |
| *Angustimassarina* | Pathotroph-Saprotroph |
| *Stemphylium loti* | Pathotroph-Saprotroph |
| *Fomitopsis pinicola* | Pathotroph-Saprotroph |
| *Caliciaceae* | Pathotroph-Saprotroph-Symbiotroph |
| *Marchandiobasidium aurantiacum* | Pathotroph-Symbiotroph |
| *Filobasidium* | Saprotroph |
| *Filobasidium magnum* | Saprotroph |
| *Laetiporus gilbertsonii* | Saprotroph |
| *Filobasidium* | Saprotroph |
| *Sporormia subticinensis* | Saprotroph |
| *Filobasidium oeirense* | Saprotroph |
| *Pyrenochaeta cava* | Saprotroph |
| *Bensingtonia ciliata* | Saprotroph |
| *Heterobasidion* | Saprotroph |
| *Trametes versicolor* | Saprotroph |
| *Skeletocutis* | Saprotroph |
| *Pleuroascus nicholsonii* | Saprotroph |
| *Perenniporia* | Saprotroph |
| *Donkioporia expansa* | Saprotroph |
| *Laetiporus* | Saprotroph |
| *Parmastomyces corticola* | Saprotroph |
| *Schizothecium inaequale* | Saprotroph |
| *Laetiporus sulphureus* | Saprotroph |
| *Phlebia centrifuga* | Saprotroph |
| *Skeletocutis chrysella* | Saprotroph |
| *Phlebia* | Saprotroph |
| *Trametes hirsuta* | Saprotroph |
| *Phanerochaete sordida* | Saprotroph |
| *Phlebia livida* | Saprotroph |
| *Phlebia tremellosa* | Saprotroph |
| *Schizophyllum commune* | Saprotroph |
| *Schizothecium vesticola* | Saprotroph |
| *Oxyporus subpopulinus* | Saprotroph |
| *Irpex lacteus* | Saprotroph |
| *Trechispora subsphaerospora* | Saprotroph |
| *Chrysosporium* | Saprotroph |
| *Pyrenochaeta* | Saprotroph |
| *Apiosordaria* | Saprotroph |
| *Zopfiella longicaudata* | Saprotroph |
| *Cercophora* | Saprotroph |
| *Phlebia margaritae* | Saprotroph |
| *Phanerochaete* | Saprotroph |
| *Antrodia serialis* | Saprotroph |
| *Thielavia* | Saprotroph |
| *Bensingtonia ciliata* | Saprotroph |
| *Bjerkandera adusta* | Saprotroph |
| *Meruliporia incrassata* | Saprotroph |
| *Auriporia aurea* | Saprotroph |
| *Podospora decipiens* | Saprotroph |
| *Pichia kluyveri* | Saprotroph-Symbiotroph |
| *Evernia prunastri* | Symbiotroph |
| *Acarosporaceae* | Symbiotroph |
| *Physciaceae* | Symbiotroph |
| *Parmeliaceae* | Symbiotroph |
| *Candelariaceae* | Symbiotroph |
| *Lecidea cancriformis* | Symbiotroph |
| *Melanelixia* | Symbiotroph |
| *Lecidea laboriosa* | Symbiotroph |
| *Melanohalea* | Symbiotroph |
| *Acarospora* | Symbiotroph |
| *Lecidea* | Symbiotroph |
| *Letharia vulpina* | Symbiotroph |
| *Elaphomyces muricatus* | Symbiotroph |
| *Placynthiella icmalea* | Symbiotroph |
| *Buellia griseovirens* | Symbiotroph |
| *Physconia isidiigera* | Symbiotroph |
| *Physconia perisidiosa* | Symbiotroph |
| *Physconia* | Symbiotroph |
| *Melanohalea elegantula* | Symbiotroph |
| *Candelaria pacifica* | Symbiotroph |
| *Placidium lacinulatum var erythrostratum* | Symbiotroph |
| *Polycauliona polycarpa* | Symbiotroph |
| *Fulgidea sierrae* | Symbiotroph |
| *Sarea* | Symbiotroph |
| *Trapeliopsis californica* | Symbiotroph |
| *Cenococcum geophilum* | Symbiotroph |
| *Placidium lacinulatum* | Symbiotroph |
| *Buellia* | Symbiotroph |
| *Phialocephala dimorphospora* | Symbiotroph |
| *Craterellus tubaeformis* | Symbiotroph |
| *Tomentella* | Symbiotroph |
| *Rhizopogon* | Symbiotroph |
| *Phaeophyscia* | Symbiotroph |
| *Hygrophorus* | Symbiotroph |
| *Caloplaca* | Symbiotroph |
| *Thelomma ocellatum* | Symbiotroph |
| *Rhizopogon brunneiniger* | Symbiotroph |
| *Cadophora* | Symbiotroph |
| *Xanthomendoza galericulata* | Symbiotroph |
| *Leptosphaerulina australis* | Symbiotroph |
| *Parmelina coleae* | Symbiotroph |
| *Rhizopogon roseolus* | Symbiotroph |
| *Pisolithus* | Symbiotroph |
| *Tomentella sublilacina* | Symbiotroph |
| *Cortinarius acutus* | Symbiotroph |
| *Lecanora* | Symbiotroph |
| *Placidium umbrinum* | Symbiotroph |
| *Leprocaulon santamonicae* | Symbiotroph |
| *Russula* | Symbiotroph |
| *Lecanora fuscobrunnea* | Symbiotroph |
| *Geopora cooperi* | Symbiotroph |
| *Rhizopogon evadens* | Symbiotroph |
| *Verrucaria* | Symbiotroph |
| *Xanthoria fallax* | Symbiotroph |
| *Aureoboletus mirabilis* | Symbiotroph |
| *Nodobryoria oregana* | Symbiotroph |
| *Elaphomyces decipiens* | Symbiotroph |
| *Waynea californica* | Symbiotroph |
| *Endocarpon* | Symbiotroph |
| *Pisolithus arhizus* | Symbiotroph |
