# Supplementary material for: Landscape Topography and Regional Drought Alters Dust Microbiomes in the Sierra Nevada of California
Source: Front Microbiol. 2022 Jun 28;13:856454. doi: 10.3389/fmicb.2022.856454 (PMC9274194; doi:10.3389/fmicb.2022.856454)
Supplement: Supplementary Table 3 — Indicator species analyses for significant fungal taxa (to genus- or species-level). Only significant p-values (p < 0.05) reported. [file Table_3.docx]

Supplementary Table 3. Indicator species analyses for significant fungal taxa (to genus- or species-level). Only significant p-values (p<0.05) reported.

| **Site** | **P-value** | **Class** | **Order** | **Family** | **Genus** | **Species** |
| --- | --- | --- | --- | --- | --- | --- |
| **SHORTHAIR** | 0.046 | Sordariomycetes | Xylariales | Xylariaceae | *Anthostomella* | *Anthostomella pinea* |
| **SHORTHAIR** | 0.013 | Lecanoromycetes | Caliciales | Caliciaceae | *Buellia* | *Buellia griseovirens* |
| **SHORTHAIR** | 0.041 | Dothideomycetes | NA | NA | *Catenulomyces* | *Catenulomyces convolutus* |
| **PROVIDENCE** | 0.024 | Tremellomycetes | Cystofilobasidiales | Cystofilobasidiaceae | *Cystofilobasidium* | *Cystofilobasidium macerans* |
| **SHORTHAIR** | 0.005 | Lecanoromycetes | Umbilicariales | Umbilicariaceae | *Fulgidea* | *Fulgidea sierrae* |
| **SHORTHAIR** | 0.036 | Dothideomycetes | Capnodiales | Teratosphaeriaceae | *Lapidomyces* | *Lapidomyces hispanicus* |
| **SHORTHAIR** | 0.009 | Saccharomycetes | Saccharomycetales | NA | *Myxozyma* | *Myxozyma melibiosi* |
| **SHORTHAIR** | 0.036 | Lecanoromycetes | Lecanorales | Parmeliaceae | *Parmelina* | *Parmelina coleae* |
| **SHORTHAIR** | 0.042 | Agaricomycetes | Polyporales | Meruliaceae | *Phlebia* | *Phlebia centrifuga* |
| **SHORTHAIR** | 0.049 | Sordariomycetes | Diaporthales | Valsaceae | *Phomopsis* | *Phomopsis quercina* |
| **SHORTHAIR** | 0.036 | Lecanoromycetes | Caliciales | Physciaceae | *Physconia* | *Physconia perisidiosa* |
| **SHORTHAIR** | 0.036 | Dothideomycetes | Pleosporales | Pleosporaceae | *Pyrenophora* | *Pyrenophora biseptata* |
| **PROVIDENCE** | 0.043 | Microbotryomycetes | Sporidiobolales | Sporidiobolaceae | *Rhodotorula* | *Rhodotorula mucilaginosa* |
| **PROVIDENCE** | 0.019 | Dothideomycetes | Pleosporales | Pleosporaceae | *Alternaria* | NA |
| **SHORTHAIR** | 0.027 | Tremellomycetes | Tremellales | Tremellaceae | *Cryptococcus* | NA |
| **PROVIDENCE** | 0.019 | Tremellomycetes | Filobasidiales | Filobasidiaceae | *Naganishia* | NA |
| **SHORTHAIR** | 0.027 | Dothideomycetes | Capnodiales | Teratosphaeriaceae | *Neocatenulostroma* | NA |
| **SHORTHAIR** | 0.008 | Dothideomycetes | Pleosporales | Phaeosphaeriaceae | *Neostagonospora* | NA |
| **SOAPROOT** | 0.029 | Microbotryomycetes | Sporidiobolales | Sporidiobolaceae | *Rhodosporidiobolus* | NA |
| **PROVIDENCE** | 0.016 | Microbotryomycetes | Sporidiobolales | Sporidiobolaceae | *Rhodotorula* | NA |
| **SJER** | 0.049 | Pezizomycetes | Pezizales | Pyronemataceae | *Trichophaea* | NA |
| **SHORTHAIR** | 0.016 | Tremellomycetes | Tremellales | Bulleribasidiaceae | *Vishniacozyma* | NA |
